# Evaluation of Compliance Issues to Anti-glaucoma Medications Before and After a Structured Interventional Program

**DOI:** 10.7759/cureus.25943

**Published:** 2022-06-14

**Authors:** Irshad A Subhan, Rawan Alosaimy, Nouf T Alotaibi, Bayan Mirza, Ghufran Mirza, Orjwan Bantan

**Affiliations:** 1 Department of Ophthalmology, King Abdullah Medical City, Makkah, SAU; 2 Department of Medicine and Surgery, College of Medicine, Umm Al-Qura University, Makkah, SAU; 3 Department of Pediatrics, Maternity and Children’s Hospital, Al-Madinah, SAU; 4 Department of Medicine and Surgery, Batterjee Medical College, Jeddah, SAU

**Keywords:** improve patient outcomes, patient education, eyedrops, anti-glaucoma medications, drug compliance, glaucoma

## Abstract

Background

Glaucoma is one of the most common eye diseases in the elderly and the major cause of irreversible vision loss worldwide. Adherence to life-long therapies is crucial to prevent glaucoma progression. The current study aims to assess the educational element and its impact on glaucoma medication compliance over short and long periods.

Methods

This was a survey-based, prospective, interventional study, conducted via interviews of all glaucoma patients presented to the Ophthalmology Center at King Abdullah Medical City (KAMC), Makkah, Saudi Arabia. To achieve the study’s aim, a questionnaire with 31 items was utilized, followed by a structured program between September 2019 to June 2021. After that, a second questionnaire was used after a one month to one year to re-evaluate the intervention. Data was automatically collected in Microsoft Excel (Microsoft Corporation, Redmond, Washington, United States) and entered into IBM SPSS Statistics for Windows, Version 22.0 (Released 2013; IBM Corp., Armonk, New York, United States) for analysis.

Results

Non-compliance was detected in 15.7% of all recruited patients (n=134). However, the non-compliance percentage dropped to 10 (7.5%) after the structured program (P=0.028). Contributing factors were low educational level, bilateral eye disease, duration of treatment more than two years, and having more than two eye treatment bottles; however, the P-value was insignificant.

Conclusions

About one-sixth of our glaucoma patients were found to be non-compliant. However, the non-compliance reduced by more than half after the structured educational program. Treatment adherence can be improved by implementing awareness and correcting the beliefs about illness and medicines, thus potentially delaying disease development.

## Introduction

Glaucoma is one of the common aging eye diseases and the major cause of irreversible vision loss in adults worldwide [1]. The global prevalence of glaucoma is 3.57% among individuals aged more than 40 years [2]. Locally, glaucoma affects 5.6% of the population aged 40 years and older in Riyadh [3]. In comparison, a higher prevalence of 17.7% is reported in a single center in the western region of Saudi Arabia [4]. 

Management of glaucoma is life-long. The main goal of the treatment is to prevent the progression of vision loss, which can only be done by lowering the intraocular pressure (IOP). Medical therapy, laser, and surgery are all available options; however, medical therapy is preferred by both ophthalmologists and glaucoma patients, as it is the safest. Therefore, adherence to life-long therapy is crucial in reducing its effect on vision [5,6]. On the other hand, sub-optimal adherence to anti-glaucoma medications is the primary cause of progressive visual field loss and eventual blindness [7,8]. 

Studies identified further interference to adherence, including poor education, lack of motivation, forgetfulness, and difficulties in drop application [9]. Besides the availability of medications at a reasonable cost, simplifying treatment regimens and interactive health education appear to be the most critical factors for improving compliance [10]. Since many of the reasons that contribute to poor adherence have to do with patient knowledge about glaucoma and familiarity with therapy, these issues could be mitigated by better or alternative communication and education strategies [11]. It has long been assumed that patient education has a positive effect. However, it is still questionable how much patient education, which improves knowledge, can improve medication adherence [11,12].

Patients' practice toward anti-glaucoma drugs varies in the literature, with recent studies reporting non-compliance rates ranging from 16% to 67.5% [5,13]. Nevertheless, the scarcity of data observed in Makkah is due to limited studies investigating this subject. Quantifying the responsible issues is crucial in improving patient care [11,14]. Hence, it is crucial to evaluate them to determine their efficacy in both long- and short-term circumstances. Therefore, this study was undertaken to investigate the pattern of compliance before and after a structured educational program among glaucoma patients at King Abdullah Medical City (KAMC), Makkah, Saudi Arabia. The current study aims to assess the educational element and its impact on glaucoma medication compliance over short and long periods.

## Materials and methods

Research design, population, and sample size determination

This survey-based, prospective interventional study was conducted via interviews between September 2019 and July 2021. It included all glaucoma patients attending the Ophthalmology Center at KAMC, Makkah, Saudi Arabia. Patients aged less than 18 years and in-patients were excluded; a total of 188 patients participated in this study.

Data collection tool and method

A modified questionnaire inspired by previously published studies was used for data collection [7,15]. Validity was assessed before distribution. Both Arabic and English languages were used in the questionnaire.

The patients were interviewed by senior medical students in a private room in the ophthalmology clinic to fill out the first questionnaire. After that, the patients were subjected to a structured program, which consisted of educational brochures about glaucoma and a video explaining the appropriate method to use eye-drop medications. Finally, patients were approached via phone calls to complete the second questionnaire to evaluate the intervention after one month to one year (see Appendix).

The information presented in the educational program brochure was collected from the American Optometric Association and the American Academy of Ophthalmology [16-18]. In addition, the educational video was created by the media department in KAMC explaining the appropriate method of applying eye drops.

Concerning questionnaires, the first questionnaire comprised 31 items, divided into three parts; Part 1 contained demographic information about the patient’s age, gender, nationality, educational level, address, and additional information if the patient uses a wheelchair, systemic diseases, and ocular disease (whether unilateral and bilateral); Part 2 included detailed information about the glaucoma status, anti-glaucoma medication, duration of the treatment, number of eye medication bottles, and number of drops the patient is applying; Part 3 included compliance data assessing four domains: awareness, knowledge, practicing difficulties, and satisfaction. The short-term assessment was done after one month, and the long-term evaluation was done after a year. The second questionnaire with five items was used to re-evaluate the compliance.

Data, generated in percentage, was used to segregate compliance and non-compliance. A percentage of 100-75 was classified as compliant and a percentage range from 50-25 was classified as non-compliant.

Ethical consideration

Approval was obtained for the study from the Institutional Review Board of KAMC, Makkah, Saudi Arabia (approval number 18-457), and the data was collected anonymously. The study participants were informed about the aim of this study and the benefits of their participation. Verbal informed consent was taken from the participants. For confidentiality, patients were identified by serial study code and initials linked to the patient’s name and medical record number (MRN) in a separate identification log sheet. 

Data analysis

After data were extracted, it was revised, coded, and fed to IBM SPSS Statistics for Windows, Version 22.0 (Released 2013; IBM Corp., Armonk, New York, United States). All statistical analysis was done using two-tailed tests. P-value less than 0.05 was statistically significant. Descriptive analysis based on frequency and percent distribution was done for all variables. Pearson's Chi-square test was used for categorical values to assess the association between the level of compliance and socio-demographic data.

## Results

General characteristics of the participants

The recruited sample consisted of 188 glaucoma patients, of whom 134 (71.3%) completed a re-evaluation of compliance (the second questionnaire). Table 1 shows the socio-demographics of the patients, distributed according to their follow-up period. More than half, 74 (55.2%), of the participants were between 51 and 70 years. The number of males, 67 (50%), was equal to females. The majority of patients were Saudis (129; 96.3%) and 104 (77.6%) were residents of Makkah city. About a third of these subjects, 40 (29.9%), had education above university level, while 37 (27.6%) were illiterate. Of the subjects, 21 (15.7%) had associated ocular diseases like cataracts, 13 (9.7%) had retinal detachment, and nine subjects (6.7%) had diabetic retinopathy. Common systemic diseases like diabetes mellitus and hypertension were found in 78 (58.2%) and 62 (46.3%) patients, respectively.

**Table 1:** Socio-demographic characteristic of glaucoma patients attending King Abdullah Medical City, Makkah, Saudi Arabia

Variables	Follow-up after 30 days	Follow-up after 12 months	
	N=50 (37.3%)	N=84 (62.7%)	Overall (n=134)
Age			
18 to 30	7 (14%)	7 (8.3%)	14 (10.4%)
31 to 50	8 (16%)	18 (21.4%)	26 (19.4%)
51 to 70	28 (56%)	46 (54.8%)	74 (55.2%)
> 70	7 (14%)	13 (15.5%)	20 (14.9%)
Gender			
Male	29 (58%)	38 (35.2%)	67 (50%)
Female	21 (42%)	46 (54.8%)	67 (50%)
Nationality			
Saudi	48 (96%)	81 (96.4%)	129 (96.3%)
Level of education			
Illiterate	16 (32%)	21 (25%)	37 (27.6%)
Middle/Primary school	8 (16%)	18 (21.5%)	26 (19.4%)
High school	12 (24%)	19 (22.6%)	31 (23.1%)
University and above	14 (28%)	26 (31%)	40 (29.9%)
Residence			
Makkah	37 (74%)	67 (79.8%)	104 (77.6%)
Using wheelchair			
Yes	9 (18%)	5 (6%)	14 (10.4%)
Ocular disease			
Cataract	1 (2%)	20 (23.8%)	21 (15.7%)
Retinal detachment	9 (18%)	4 (48%)	13 (9.7%)
Diabetic retinopathy	4 (8%)	5 (6%)	9 (6.7%)
Ocular trauma	2 (4%)	2 (2.4%)	4 (3%)
Others	1 (2%)	3 (3.6%)	4 (3%)
Systemic disease			
HTN	24 (48%)	38 (45.2%)	62 (46.3%)
DM	28 (56%)	50 (59.5%)	78 (58.2%)
Heart disease	9 (18%)	8 (9.5%)	17 (12.7%)
Mental/neurological	0	4 (4.8%)	4 (3%)
Arthritis	9 (18%)	4 (4.8%)	13 (9.7%)
Asthma/allergy	2 (4%)	0	2 (1.5%)
Oncology	3 (6%)	2 (1.2%)	4 (3%)
Renal disease	3 (6%)	1 (1.2%)	4 (3%)
Others	1 (2%)	2 (2.4%)	3 (2.2%)

Glaucoma and anti-glaucoma medication

Table 2 illustrates factors related to anti-glaucoma medications distributed according to the follow-up period. Eighty-nine subjects (66.4%) were on anti-glaucoma medications in both eyes. 43 (32.1%) of the patients were on medications for more than six years. 37 (27.6%) and 44 (32.8%) of these patients were on two and three bottles, respectively. Moreover, only 77 (57.5%) of the patients applied one drop from each bottle.

**Table 2:** Disease pattern and anti-glaucoma medication use among glaucoma patients attending King Abdullah Medical City, Makkah, Saudi Arabia

Variables	Follow-up after 30 days	Follow-up after 12 months	
	N=50 (37.3%)	N=84 (62.7%)	Overall (n=134)
Eye treated			
Bilateral	32 (64%)	57 (67.9%)	89 (66.4%)
Unilateral	18 (36%)	27 (32.1%)	45 (33.6%)
Duration of treatment			
<6 months	7 (14%)	7 (8.3%)	14 (10.4%)
>6 months	10 (20%)	17 (20.2%)	27 (20.1%)
>2 years	12 (24%)	21 (25%)	33 (24.6%)
>4 years	5 (10%)	12 (14.3%)	17 (12.7%)
>6 years	16 (32%)	27 (32.1%)	43 (32.1%)
Number of eye medication bottles currently taken.			
1 eye bottle	11 (22%)	14 (16.7%)	25 (18.7%)
2 eye bottles	7 (14%)	30 (35.7%)	37 (27.6%)
3 eye bottles	18 (36%)	26 (31%)	44 (32.8%)
4 eye bottles	10 (20%)	10 (11.9%)	20 (14.9%)
5 eye bottles	4 (8%)	4 (4.8%)	8 (6%)
Number of drops applied from each bottle.			
1 drop	29 (58%)	48 (57.1%)	77 (57.5%)
2 drops	17 (34%)	29 (34.5%)	46 (34.3%)
3 drops	2 (4%)	5 (6%)	7 (5.2%)
More than 3 drops	2 (4%)	2 (2.4%)	4 (3%)

Compliance data

Table 3 illustrates compliance data before the educational program assessing four aspects: awareness and knowledge, practice, difficulties, and satisfaction with anti-glaucoma medication.

Awareness/Knowledge

A total of 91 subjects (67.9%) were aware that applying more than one drop is ineffective. Sixty-six subjects (49.3%) believed that the condition's progression became stable. In addition, 73 subjects (54.5%) believed glaucoma could not be cured, while 72 subjects (53.7%) were aware that glaucoma is a chronic condition and requires lifelong medication.

Practice

A total of 103 subjects (76.9%) used eye medications during fasting, while 30 subjects (22.4%) believed that using eye medication during fasting would break their fast. Of the total subjects, 86 (64.2%) confessed to using eye medication regularly. Regarding the level of compliance with doctors’ instructions, 44 subjects (32.8%) had 100% compliance, while the majority, 69 (51.5%) of the patients, had 75% compliance. On the other hand, fair and poor compliance was detected among 15 (11.2%) and six (4.5%), respectively. Ninety-six subjects (71.6%) had instructions about using eye medications appropriately. While 99 subjects (73.9%) were applying the eye drops by themselves, 53 patients (39.6%) needed help applying eye drops, usually by their family members (57; 42.5%).

Difficulties

Fourty-four subjects (32.8%) faced difficulties applying eye drops. Seventy-four subjects (55.2%) had a burning sensation, 34 patients (25.4%) had itching, 27 patients (20.1%) had redness of the eye, one patient (0.7%) reported shortness of breath, and three patients (2.2%) reported headaches. The most frequently reported reason for not using medications regularly was forgetfulness in 39 subjects (29.1%), followed by laziness in 21 (15.7%).

Satisfaction

A total of 128 subjects (95.5%) were comfortable using their medication and 130 subjects (97%) got their medication from the hospital pharmacy.

**Table 3:** Compliance data before educational program assessing four aspects among glaucoma patients attending King Abdullah Medical City, Makkah, Saudi Arabia

Variables	Overall (n=134)
Knowledge/Awareness	
Do you think applying more than one drop is more effective?	
No	91 (67.9%)
Do you know about the progress of glaucoma?	
The condition becomes better	38 (28.4%)
The condition becomes stable	66 (49.3%)
The condition becomes worse	30 (22.4%)
Do you think glaucoma can be cured?	
No	73 (54.5%)
Do you know glaucoma is chronic and needs lifetime medication?	
Yes	72 (53.7%)
Practice	
Do you use medications during fasting?	
Yes	103 (76.9%)
Do you think using eye medication during fasting will break your fast?	
Yes	30 (22.4%)
Are you using the eye medication regularly?	
Yes	86 (64.2%)
How much are compliant to your doctor instructions?	
Excellent (100%)	44 (32.8%)
Good (75%)	69 (51.5%)
Fair (50%)	15 (11.2%)
Poor (25%)	6 (4.5%)
Have you been instructed the way of using eye medications?	
Yes	96 (71.6%)
Do you put eye drops by yourself?	
Yes	99 (73.9%)
Do you usually need help?	
Yes	53 (39.6%)
If yes who helps:	
Son/daughter	38 (28.4%)
Wife/husband	15 (11.2%)
Maid	6 (4.5%)
Other	6 (4.5%)
Difficulties	
Do you face difficulty in applying eye drops?	
Yes	44 (32.8%)
Do you suffer any symptoms while using medications?	
Burning	74 (55.2%)
Eye Redness	27 (20.1%)
Itching	34 (25.4%)
Dryness	16 (11.9%)
Tearing	9 (6.7%)
Blurred vision	10 (7.5%)
Bitter taste	11 (8.2%)
Headache	3 (2.2%)
Shortness of breath	1 (.7%)
If poor compliance, what are the reasons for missing the dose:	
Forgetfulness	39 (29.1%)
Carrying a lot of medication	5 (3.7%)
Sleep disturbance	1 (.7%)
Traveling	2 (1.5%)
Unavailability of drugs (empty bottles of drugs)	12 (9%)
Laziness	21 (15.7%)
Satisfaction	
Are you comfortable after using your medication?	
Yes	128 (95.5%)
Do you get your medication from hospital pharmacy?	
Yes	130 (97%)

Second questionnaire

Table 4 illustrates the evaluation of compliance issues after the educational program. A total of 130 subjects (97.0%) benefited from the exercise in using their medications. Also, 97 subjects (72.4%) understood the information in the brochure. The majority, 124 subjects (92.5%), felt comfortable with their eye medications. As for the re-evaluation of compliance to doctor instructions, the majority, 71 subjects (53%) had 100% compliance (excellent), 53 subjects (39.6%) had 75% compliance (good), while six (4.5%) had 50% compliance (fair), and four subjects (3%) had 25% compliance (poor).

**Table 4:** Evaluation of compliance issues after structured interventional program among glaucoma patients attending King Abdullah Medical City, Makkah, Saudi Arabia (n=134)

Variables	Follow-up after 30 days	Follow-up after 12 months	
	N=50 (37.3%)	N=84 (62.7%)	Overall (n=134)
Was our explanation and presentations helpful in using your medications:			
Yes	48 (96%)	82 (97.6%)	130 (97%)
Are you feel comfortable using your medications?			
Yes	32 (64%)	65 (77.4%)	124 (92.5%)
Were you able to understand the information in brochure?			
Yes	48 (96%)	76 (90.5%)	97 (72.4%)
Compliance with your doctor instructions:			
Excellent (100%)	30 (60%)	41 (48.8%)	71 (53%)
Good (75%)	16 (32%)	37 (44%)	53 (39.6%)
Fair (50%)	4 (8%)	2 (2.4%)	6 (4.5%)
Poor (25%)	0	4 (4.8%)	4 (3%)

Compliance before and after the intervention with respect to sociodemographics

Twenty-one subjects (15.7%) had poor compliance before the intervention in comparison to only 10 subjects (7.5%) after the intervention with P value 0.028, which was statistically significant. As per the compliance data with regards to the time of follow-up, a high percentage of poor compliance was detected among those who had followed up after one year (60%) compared to (40%) of those who had followed up after a month (P=0.855). Poor compliance was higher among those who had bilateral eye disease (80%) in comparison to unilateral eye disease (20%) (P=0.344). The compliance data with regards to duration of the treatment showed that 70% of the patients who had treatment for more than two years had poor compliance, compared to 30% who were treated for less than two years (P=0.966). Moreover, 80% of poor compliant patients were using more than two eye drops compared to 20% of the patients who were using less or equal to two eye bottles (P=0.083). As for the compliance data about age, gender, and systemic disease presence, poor compliance was detected among the subgroups equally (P=0.148, 1.00, and 0.074, respectively) (Table 5).

**Table 5:** Relation between compliance and sociodemographics of patients after structured interventional program Note: * significant p, at the 0.05 level

Variables	Compliance after the educational program			
	Total	Good Compliance	Non-compliance	P-value
Compliance before the educational program				
Good compliance	113 (84.3%)	107 (86.3%)	6 (60%)	*0.028
Poor compliance	21 (15.7%)	17 (13.7%)	4 (40%)	
Follow-up period duration				0.855
After (30 days)	50 (37.3%)	46 (37.1%)	4 (40%)	
After (12 months)	84 (62.7%)	78 (62.9%)	6 (60%)	
Age				
<50 years	40 (29.9%)	35 (28.2%)	5 (50%)	.148
>50	94 (70.1%)	89 (71.8%)	5 (50%)	
Gender				
Male	67 (50%)	62 (50%)	5 (50%)	1.00
Female	67 (50%)	62 (50%)	5 (50%)	
Nationality				
Saudi	129 (96.3%)	119 (96%)	10 (100%)	0.518
Non-Saudi	5 (3.7%)	5 (4%)	0	
Level of education				
High school	31 (23.1%)	29 (23.4%)	2 (20%)	0.394
Illiterate	37 (27.6%)	35 (28.2%)	2 (20%)	
Primary/Middle school	26 (19.4%)	22 (17.7%)	4 (40%)	
University and above	40 (29.9%)	38 (30.6%)	2 (20%)	
Residence				
Makkah	104 (77.6%)	98 (79%)	6 (60%)	0.165
Outside Makkah	30 (22.4%)	26 (21%)	4 (40%)	
Systematic disease				
Yes	99 (73.9%)	94 (75.8%)	5 (50%)	0.074
Not known	35 (26.1%)	30 (24.2%)	5 (50%)	
Eye treated				
Bilateral	89 (66.4%)	81 (65.3%)	8 (80%)	0.344
Unilateral	45 (33.6%)	43 (34.7%)	2 (20%)	
Duration of treatment				
more than two years	93 (69.4%)	86 (69.4%)	7 (70%)	0.966
less than two years	41 (30.6%)	38 (30.6%)	3 (30%)	
Number of eye medication bottles				
more than two bottles	62 (46.3%)	60 (48.4%)	2 (20%)	0.083
less than two bottles	72 (53.7%)	64 (51.6%)	8 (80%)	
Difficulty in applying				
Yes	90 (67.2%)	41 (33.1%)	3 (30%)	0.843
No	44 (32.8%)	83 (66.9%)	7 (70%)	

## Discussion

Poor adherence to topical glaucoma medication is a worldwide issue. It is not limited to ophthalmological conditions, as only 50-70% of prescribed dosages are taken for other chronic medical conditions such as hypertension. In addition, poor adherence to medical therapy is costly to both the patient and the healthcare system, resulting in higher resource consumption [19]. According to a recent Cochrane systematic review, there is insufficient evidence to advocate for any specific intervention for glaucoma medication adherence. However, an improved understanding of the most fundamental factors may enhance interventions [19]. To the best of our knowledge, this is the first study investigating the level of adherence to glaucoma medication before and after an educational program in Saudi Arabia that provides preliminary data to improve glaucoma medication adherence.

Approximately one-sixth (15.7%) of glaucoma patients were not adherent to their medications in this study. The rate noted in the current study is less than the earlier studies done in Saudi Arabia. In 2012, the non-adherence rate in Riyadh was 19.4% [7], whereas, in 2017, it was 27.4% and 27.8% in Riyadh and Jeddah, respectively [6,20]. Internationally, nearly similar data were reported in Korea (27.4%) and the United States (27%) [21,22]. In contrast, higher rates was reported in other countries: 39.2% in Brazil [23], 43.8% in Ethiopia [24], 49% in India [13], 50% in Israel [25], 51.6% in United Kingdom [26], 53.6% in Egypt [27] and 60% in Turkey [28]. This wide variation in the non-adherence rate can be justified partly by the variability of study methodologies (subjective or objective) and non-uniform compliance definitions. The adherence to glaucoma medication could improve by identifying and addressing the responsible barriers [6].

The current study evaluated the importance of educational status as it critically influences patients' compliance with glaucoma medication. Our study showed that the rate of non-adherence to glaucoma medication dropped significantly to 7.5% after the structured interventional program (P=0.028). The result was statically significant. Similarly, a relatively large randomized control trial (RCT) conducted in the United Kingdom demonstrated that a personalized patient care package that includes educational sessions enhanced prescription collection after 12 months and significantly reduced IOP variations and clinical management adjustments after 24 months [29]. This result confirms that an accurate understanding of the condition and constant reminders are fundamentally linked to compliance, and lack of awareness about their disease's progression and the permanent component of glaucoma blindness can lead to poor treatment adherence.

Since financial implications could be the reason for poor adherence to glaucoma medications, it is worth mentioning that the evaluated glaucoma patients are exceptional, in which ophthalmic services and drugs are either free or paid by the insurance company in most cases for Saudi residents. As a result, the expense of medication is less likely to be a barrier in this demographic, resulting in non-adherence to glaucoma medical therapy. However, despite this free healthcare system, the non-adherence of 15.7% of glaucoma patients must be investigated further to find a solution [6].

Although demographics and socioeconomic variables were not significantly associated with adherence to glaucoma treatment, a pattern was observed in the current study. A longer follow-up period and longer duration of glaucoma were associated with the poor adherence rate after the educational program. This finding is comparable to a prior study that assessed adherence over four years of follow-up as the prevalence of poor adherence increased over time, highlighting the known concern that adherence to chronic medication is a widespread problem among glaucoma patients [30]. To address this issue, Newman-Casey et al. found that if patients are over 70% adherent during their first year of treatment, they are unlikely to experience decreased adherence in the following years. As a consequence, to improve longer-term adherence, it may be vital to keep patients come back for more frequent visits to eye care providers for the first two years after starting glaucoma medications in order to allow more time for discussions about medication adherence issues or the consideration of alternative treatments if adherence declines [30].

Limitation

The current study design, like most research, has several limitations. To begin, our study was a survey-based, prospective, interventional study in study design, and patients were interviewed to complete the questionnaire. Therefore, the subjective nature of the study and the need to rely on patients' memory could be biased. However, the study sample is diverse in age, gender, and educational level; therefore, we do not believe this will impact the overall conclusion. In addition, this is a single-center experience, and the sample size was small; thus, the study's results may have been limited. Furthermore, objective studies in different centers are recommended.

## Conclusions

Around one-sixth of our glaucoma patients were found to be non-compliant. However, the non-compliance fell by more than half after the structured educational program. These findings suggest that the role of the physician is vital in patient education regarding administering glaucoma drops, correcting wrong beliefs about illness and medicines, and adjusting treatment to their healthcare needs and can improve adherence to ocular hypotensive therapy, potentially delaying disease development.

## Appendices

Questionnaire I

A. Personal information

1. Age:

☐ 18-30    

☐ 31-50  

☐ 51-70  

☐ Above 70 years

2. Gender:

☐ Male                  

☐ Female

3. Nationality:

☐ Saudi

☐ Non-Saudi

4. Residence:

☐ Makkah

☐ Outside Makkah

5. Educational level:

☐ Illiterate

☐ Middle/Primary school

☐ High school

☐ University and above

6. Are you using a wheelchair:

☐ Yes

☐ No

7. Chose any ocular disease you have:

☐ Glaucoma

☐ Cataract

☐ Retinal detachment

☐ Diabetic retinopathy

☐ Ocular trauma

☐ Others:

8. Choose any systemic disease you have:

☐ Heart disease

☐ HTN

☐ DM

☐ Arthritis

☐ Oncology

☐ Mental and neurological disease

☐ Others:

B. Topical ophthalmic medication

1. Eye treated:

☐ Unilateral

☐ Bilateral

2. Duration of treatment:

☐ <6 month

☐ >6 month

☐ >2 years

☐ >4 years

☐ >6 years

3. Number of bottles of eye medication you are using:

☐ 1 eye bottle

☐ 2 eye bottles

☐ 3 eye bottles

☐ 4 eye bottles

☐ 5 eye bottles

4. Number of eye drops you are applying from each bottle:

☐1 drop

☐ 2 drops

☐ 3 drops

☐ More than 3 drops

C. Compliance data

a. Awareness/knowledge

1. Do you think applying more than 1 drop is more effective:

☐ Yes

☐ No

2. Do you know about the progress of glaucoma:

☐ The condition becomes better

☐ The condition becomes stable

☐ The condition becomes worse

3. Do you think glaucoma can be cured:

☐ Yes

☐ No

4. Do you know glaucoma is chronic and needs lifetime medication:

☐ Yes

☐ No

b. Practice

1. Do you use medications during fasting:

☐ Yes

☐ No

2. Do you think using eye medication during fasting will break your fast:

☐ Yes

☐ No

3. Do you comply with your doctor's instructions:

☐ 100 %

☐ 75 %

☐ 50 %

☐ 25 %

4. Do you put eye drops by yourself:

☐ Yes

☐ No

5. Have you been instructed on the way to use eye medications:

☐ Yes

☐ No

6. Do you usually need help:

☐ Yes

☐ No

7. If yes, who helps:

☐ Son/daughter

☐ Wife/husband

☐ Maid

☐ Other:

c. Difficulties

1. Do you face difficulty in applying eye drops:

☐ Yes

☐ No

2. Do you suffer any symptoms while using medications:

☐ Burning

☐ Eye Redness

☐ Itching

☐ Dryness

☐Tearing

☐ Blurred vision

☐ Bitter taste

☐ Headache

☐ Shortness of breath

3. Are you using the eye medication regularly:

☐ Yes

☐ No

4. If no, what are the reasons for missing the dose:

☐ Forgetfulness

☐ Carrying a lot of medication

☐ Discomfort

☐ Cost of medication

☐ Traveling

☐ Unavailability of drugs (empty bottles of drugs)

☐ Laziness

5. Are you comfortable after using your medication:

☐ Yes

☐ No

6. Do you get your medication from the hospital pharmacy:

☐ Yes

☐ No

7. Do you get your medication through insurance:

☐ Yes

☐ No

Questionnaire II

1. Was our explanation and presentations helpful in using your medications:

 Yes

 No

2. If yes, How much much:

 80%-100%

 50%-70%

 20%-40%

 Less than 20 %

3. Are you feel comfortable using your medications?

 Yes

 No

4. Were you able to understand the information in the brochure?

 Yes

 No

5. Compliance to your doctor instructions:

 100%

 75%

 50%

 25%
